# Reply: Birth rate decline, COVID-19 pandemic, and vaccination programmes

**DOI:** 10.1093/hropen/hoae068

**Published:** 2024-11-12

**Authors:** Tomáš Sobotka, Maria Winkler-Dworak, Kryštof Zeman

**Affiliations:** Vienna Institute of Demography, Austrian Academy of Sciences, Vienna, Austria; Vienna Institute of Demography, Austrian Academy of Sciences, Vienna, Austria; Vienna Institute of Demography, Austrian Academy of Sciences, Vienna, Austria

Sir,

We would like to thank Daungsupawong and Wiwanitkit (2024) for their interest in our recent article in *Human Reproduction Open* (Winkler-Dworak *et al.*, 2024) and for raising several important questions, on which we would like to comment.

Daungsupawong and Wiwanitkit’s letter suggests that the population-level data used in our study ‘could obscure significant demographic trends that are crucial for comprehending variations in the fertility rate’ (Daungsupawong and Wiwanitkit, 2024). Our study (Winkler-Dworak *et al.*, 2024) explored birth trends during the COVID-19 pandemic using population-level data with three main strengths: (i) it includes all births in each country, (ii) it covers 26 countries across four continents, and (iii) it looks at birth trends on a finer scale of months rather than years. However, the broad country coverage and the granular time scale also have limitations, as the available data do not allow further disaggregation. We refer to other studies detailing how birth trends varied by age, birth order, and for specific sub-populations during the pandemic. Their findings cannot be easily summarized here, but one conclusion stands out: disadvantaged populations, especially migrants, have often displayed more negative trends in birth rates (especially sharper and longer-lasting falls) during the pandemic than other population groups (Bailey *et al.* 2023; González and Trommlerová, 2024).

Furthermore, Daungsupawong and Wiwanitkit’s letter argues that ‘it is still unknown how exactly these factors [*ie, the factors identified in our research*] influence birth rates. The article discusses inflation and institutional trust, but not psychological and emotional factors that could also influence family planning’. (Daungsupawong and Wiwanitkit, 2024). The use of monthly aggregate-level data covering all births across many countries limited the number of factors and variables included in our model. Nonetheless, we carefully modelled the associations of these factors with birth trends, drawing on past research discussing potential pathways and mechanisms. We included interactions between covariates (especially with the level of trust in the government), looked at cross-country differences in birth rate trends, and modelled the factors associated with birth rates in specific sub-periods. By doing so, we made important inferences. For instance, we were able to rule out any ‘direct’ impact of vaccination on birth rates and concluded that a temporary dip in birth rates observed in most countries during the initial course of vaccination was related to the behavioural response among some couples who preferred avoiding pregnancy during vaccination (Winkler-Dworak *et al.*, 2024). However, a yet deeper exploration of specific mechanisms requires an entirely different set-up, using survey data and experimental studies. An emerging line of research shows that besides the ‘objective’ factors, worries, uncertainties, and expectations about the future have a strong impact on reproductive intentions and actual fertility decisions (Vignoli *et al.*, 2020; Ivanova and Balbo, 2024).

In addition, Daungsupawong and Wiwanitkit (2024) call for a deeper understanding of the underlying factors affecting people’s attitudes towards starting a family, highlighting the role of shifting health messaging and trust in public health care systems. Our research has indeed partly addressed these issues, to the extent our data and analytical setup allowed us. We have identified trust in government as an important factor influencing the relationship between policy interventions and fertility during the COVID-19 pandemic. We also argued that the extensive economic and family support policies that had been launched in the early phases of the pandemic limited the disruption that the pandemic and the associated containment measures had on people’s everyday lives. This allowed the birth rates to stabilize or even recover in most of the analysed countries during 2021 (Winkler-Dworak *et al.*, 2024).

Finally, Daungsupawong and Wiwanitkit (2024) call for research ‘on tracking birth rates for extended durations to investigate if the present patterns indicate lasting shifts or stay constant as communities adapt to the post-COVID-19 environment’ and suggests studying which ‘social support programs (…) work best to encourage higher birth rates in the future’. We hope that our study will stimulate further in-depth research investigating the intricate links between health and economic shocks, policy responses, and birth rates. Many studies have already been published on ‘pandemic births’, including research on changes in fertility intentions and desires (see especially Nitsche *et al.*, 2024). Likewise, many studies analyse the impact of social and family policies, showing that financial benefits, childcare availability, and parental leave policies often have a modest positive impact on fertility, especially when they are relatively stable and form consistent policy ‘packages’ (Luci-Greulich and Thévenon, 2013; Sobotka *et al.*, 2019). However, even this rapidly growing evidence base makes it difficult to disentangle the pandemic’s causal impact on births due to multiple crises and shocks unfolding simultaneously (Nitsche *et al.*, 2024). Past evidence shows that birth rates never remain constant for long, even in countries with extensive family and social policy support. In the face of economic uncertainty and emerging global threats and conflicts, birth rates continued falling across most of the higher-income countries in 2023 and in the first half of 2024.

## References

[hoae068-B1] Bailey MJ , Currie J, Schwandt H. The COVID-19 baby bump in the United States. Proc Natl Acad Sciences USA 2023;34:e2222075120. 10.1073/pnas.2222075120 PMC10450661 37582121

[hoae068-B2] Daungsupawong H , Wiwanitkit V. Birth rate decline, COVID-19 pandemic and vaccination programmes. Human Reproduction Open 2024;2024:hoae067. 10.1093/hropen/hoae067 PMC11590251 39600576

[hoae068-B3] González L , Trommlerová S. The impact of COVID-19 on fertility in Spain. AEA Papers and Proceedings 2024;114:381–386.

[hoae068-B4] Ivanova K , Balbo N. Societal pessimism and the transition to parenthood: a future too bleak to have children? Popul Dev Rev 2024;2:323–342.

[hoae068-B5] Luci-Greulich A , Thévenon O. The impact of family policies on fertility trends in developed countries. Eur J Population 2013;29:387–416.

[hoae068-B6] Nitsche N , Wilde J, Myrskylä M. Pandemic babies: the Covid‐19 pandemic and its impact on fertility and family dynamics. Popul Dev Rev 2024;S1:1–446.

[hoae068-B7] Sobotka T , Matysiak A, Brzozowska Z. *Policy Responses to Low Fertility: How Effective are They?* UNFPA, 2019. https://www.unfpa.org/publications/policy-responses-low-fertility-how-effective-are-they (31 October 2024, date last accessed).

[hoae068-B8] Vignoli D , Bazzani G, Guetto R, Minello A, Pirani E. Uncertainty and narratives of the future: a theoretical framework for contemporary fertility. In: Schoen R (ed). Analyzing Contemporary Fertility. Cham, Germany: Springer, 2020, 25–47.

[hoae068-B9] Winkler-Dworak M , Zeman K, Sobotka T. Birth rate decline in the later phase of the COVID-19 pandemic: the role of policy interventions, vaccination programmes, and economic uncertainty. Hum Reprod Open 2024;2024:hoae052. 39345877 10.1093/hropen/hoae052 PMC11438547

